# Horseradish Peroxidase Labelled-Sandwich Electrochemical Sensor Based on Ionic Liquid-Gold Nanoparticles for *Lactobacillus brevis*

**DOI:** 10.3390/mi12010075

**Published:** 2021-01-12

**Authors:** Le Zhao

**Affiliations:** Key Laboratory of Dairy Science, Ministry of Education, Northeast Agricultural University, Harbin 150030, China; zhaole@neau.edu.cn

**Keywords:** immunosensor, electrodeposition, *Lactobacillus brevis*, ionic liquid, beer, cyclic voltammetry

## Abstract

*Lactobacillus brevis* is the most common bacteria that causes beer spoilage. In this work, a novel electrochemical immunosensor was fabricated for ultra-sensitive determination of *L. brevis.* Gold nanoparticles (AuNPs) were firstly electro-deposited on the electrode surface for enhancing the electro-conductivity and specific surface area. Ionic liquid was used for improving the immobilization performance of the immunosensor. After optimization, a linear regression equation can be observed between the ∆_current_ and concentration of *L. brevis* from 10^4^ CFU/mL to 10^9^ CFU/mL. The limit of detection can be estimated to be 10^3^ CFU/mL.

## 1. Introduction

Beer is one of the most popular drinks in the world. The specific physical and chemical properties of beer, such as low temperature anaerobic environment, low pH (4.2–4.4) and hop bitter substances, can resist the proliferation of general microorganisms. In spite of this, there are still some acid resistant, hop resistant and anaerobic microorganisms in the brewery environment [1,2,3]. They make use of the intermediate metabolites and autolysates of yeast and bring harm to beer production. Among them, the most destructive to beer are some gram-positive bacteria such as *Lactobacillus* and *Pediococcus*. *Lactobacillus brevis* is the most common bacteria isolated from spoilage beer, which causes more than half of beer spoilage problems. It is also one of the beer spoilage bacteria which has been studied deeply [4,5,6].

Culture method and biochemical microtubule fermentation method are the most commonly used. Their advantages are convenience and low cost. Their disadvantages are that they take a long time, generally about a week, the precision is not high and the microbial pollution in the production process cannot be controlled in time [7,8,9,10,11]. Adenosine triphosphate (ATP) bioluminescence rapid detection has been applied in public health detection of food industry and pharmaceutical industry. The fluorescence intensity of the reaction is directly proportional to the amount of ATP, so the amount of ATP or microorganism on the membrane can be quantitatively detected according to the standard curve [12,13,14,15,16,17,18,19,20]. Since this method is based on intracellular ATP, it is only suitable for microbial detection in relatively clean sake and finished beer. Enzyme linked immunosorbent assay or enzyme-linked immunosorbent assay (ELISA) can be used to detect low levels of antigen. However, ELISA is actually an optical measurement, and it has some disadvantages in use. These disadvantages require a large, power intensive light source, detector and monochromator. Moreover, the color of the sample will produce potential false signals.

Electrochemical immunosensor is a molecular recognition element based on antigen-antibody reaction [21,22,23]. The concentration signal of a certain chemical substance is transformed into corresponding electrical signal through the sensor element. Electrochemical immunosensor has many advantages, such as good selectivity, variety, low cost and online application. It can be widely used in medical treatment, food analysis, industrial production and environmental detection [24,25,26]. This communication demonstrates the electrochemical assay developed for the detection of *L. brevis*. The immunosensor fabrication has involved using gold nanoparticles (AuNPs) to enhance the immobilization ability. AuNPs have been widely used for immunosensor fabrication due to their excellent conductivity for enhancing the signal [27]. In addition, ionic liquid and chitosan have been used for further enhancing the stability of antibody due to the binding and blanketing effect [28,29]. Especially, the bioactivity of biospecies could be maintained and their electrochemical activity could be promoted in ionic liquid. The proposed electrochemical immunosensor showed excellent sensing performance towards the *L. brevis* detection.

## 2. Materials and Methods

### 2.1. Reagents and Instrument

*Escherichia coli* (*E. coli*, CICC 10003), *Staphylococcus aureus* (*S. aureus*, CICC 21600), *Bacillus subtilis* (*B. subtilis*, CICC 10028) and *L. brevis* (CICC 20014) were purchased from China Center of Industrial Culture Collection, Beijing, China. Anti-*L. brevis* and horseradish peroxidase (HRP)-labeled anti-*L. brevis* were purchased from ChinaPeptides Co. Ltd., Shanghai, China. HAuCl_4_, 1-Butyl-3-methylimidazolium hexafluorophosphate (ILs), chitosan and thionine were purchased from 9dingchem Co. Ltd., Shanghai, China. All other chemicals were analytical grade and used without further purification.

All electrochemical experiments were conducted at a CHI760E electrochemical working station. A typical three-electrodes system was used, including a glassy carbon electrode (GCE), a Pt wire and an Ag/AgCl (3 M KCl) electrode.

### 2.2. Preparation of Microbial Sample

All microbes were grown at 37 °C in nutrient broth. Cells were harvested in late exponential growth phase by centrifugation (4025× *g* for 20 min) and washed using phosphate buffer saline (PBS). After removal of the supernatant fluid, the pellets were resuspended in 10 mL PBS. The density of the *L. brevis* suspension was determined to be 10^10^ CFU/mL. The *L. brevis* was inactivated 12 h at room temperature by 0.4% formaldehyde and stored at 4 °C until used. The suspension was diluted in 0.9% NaCl solution to produce the desired final concentration of *L. brevis* for experiments.

### 2.3. Preparation of Electrochemical Immunosensor

**Figure 1:**
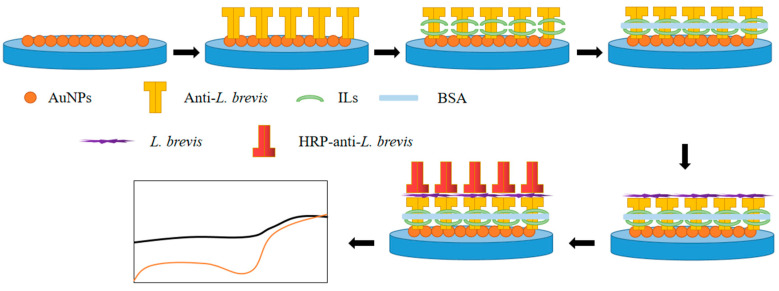
Scheme of preparation of *Lactobacillus brevis* immunosensor.

A GCE was firstly polished using Al_2_O_3_ slurry and washed by water and ethanol. Then, AuNPs were electro-deposited on the GCE by reduction of HAuCl_4_. Typically, GCE was inserted into 20 mL of 10 mg/L HAuCl_4_ solution (containing 1% HCl). Then, a cyclic voltammetry scan between −1 to 1 V at a scan rate of 10 mV/s was conducted for two cycles. After electro-deposition, the GCE was rinsed by water and ethanol and dried at room temperature. The AuNPs deposited electrode was denoted as Au/GCE. Two micrograms per litre of anti-*L. brevis* (1:200 diluted in 0.1 M PBS, pH 7.4) were coated on the above electrode and stored at 4 °C for 12 h. Five micrograms per litre of 1% (*v*/*v*) ILs or 1% chitosan (CS) was dip coated on the above electrode surface and dried at room temperature. The electrode was washed gently with PBS to remove excess antibody. Then, the electrode was immersed into a bovine serum albumin (BSA) solution (*w*/*w*, 0.25%) for blocking all active sites. The modified immunosensors were denoted as ILs/anti-*L. brevis*/Au/GCE or CS/anti-*L. brevis*/Au/GCE. The scheme of preparation of the immunosensor is shown in Figure 1.

### 2.4. Electrochemical Detection of L. brevis

Five micrograms per litre of *L. brevis* was dropped onto the ILs/anti-*L. brevis*/Au/GCE and incubated at 35 °C for half an hour and rinsed by PBS. The electrode was denoted as *L. brevis*/ILs/anti-*L. brevis*/Au/GCE. Then, 5 μL of HRP-anti-*L. brevis* was coated on the above electrode and then inserted into a 0.1 M ABS (pH 6.5) with 1 mM thionine and 0.5 mM H_2_O_2_. CV has been used for sensing analysis. The reduction peak before and after the immune reaction has been used as an indicator. All electrochemical measurements were repeated at least five times to ensure the reproducibility.

## 3. Results and Discussion

**Figure 2:**
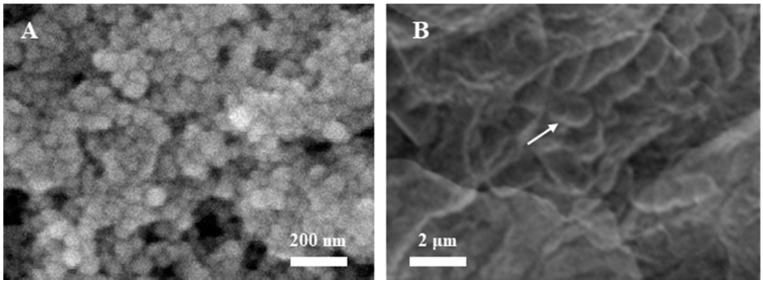
SEM image of (**A**) electro-deposited gold nanoparticles (AuNPs) and (**B**) *L. brevis*/ILs/anti-*L. brevis*/Au/GCE.

Electrochemical deposition of AuNPs can improve the performance of the immunosensor and enhance the immobilization ability of the electrode surface. Figure 2A shows the surface of GCE deposited with AuNPs. It can be seen from the figure that there are about 30 nm AuNPs on the surface of the electrode. The size of the nanoparticles is uniform, which ensures the repeatability of the immunosensor [30]. Figure 2B shows the surface of the *L. brevis*/ILs/anti-*L. brevis*/Au/GCE. The immobilization of antibody showed the coverage of the AuNPs, while the *L. brevis* was absorbed on the electrode surface.

**Figure 3:**
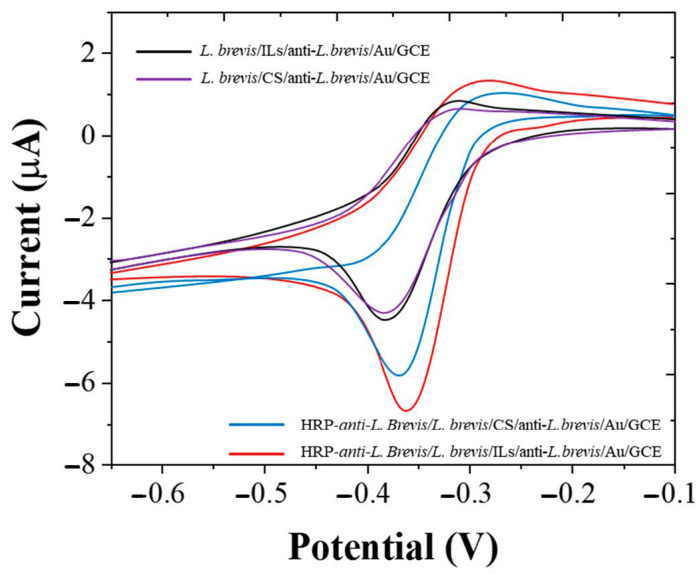
CVs of *L. brevis*/ILs/anti-*L. brevis*/Au/GCE, *L. brevis*/CS/anti-*L. brevis*/Au/GCE, HRP-anti-*L. brevis/L. brevis*/ILs/anti-*L. brevis*/Au/GCE and HRP-anti-*L. brevis/L. brevis*/CS/anti-*L. brevis*/Au/GCE.

ILs and CS are two substances that are often used to improve the stability of immunosensors. This study compared the effects of the two substances. Figure 3 shows the CV of *L. brevis*/ILs/anti-*L. brevis*/Au/GCE and *L. brevis*/CS/anti-*L. brevis*/Au/GCE before and after immune response. It can be seen from the figure that *L. brevis*/ILs/anti-*L. brevis*/Au/GCE can reduce more H_2_O_2_ after immune reaction, indicating that more HRP-anti-*L. brevis* is loaded on the electrode surface. The results show that ILs can provide an excellent microenvironment for microorganisms [31,32,33], and that the loaded substances can maintain high bioactivity. Therefore, ILs was selected as the stabilizer of immune sensor in the follow-up work.

**Figure 4:**
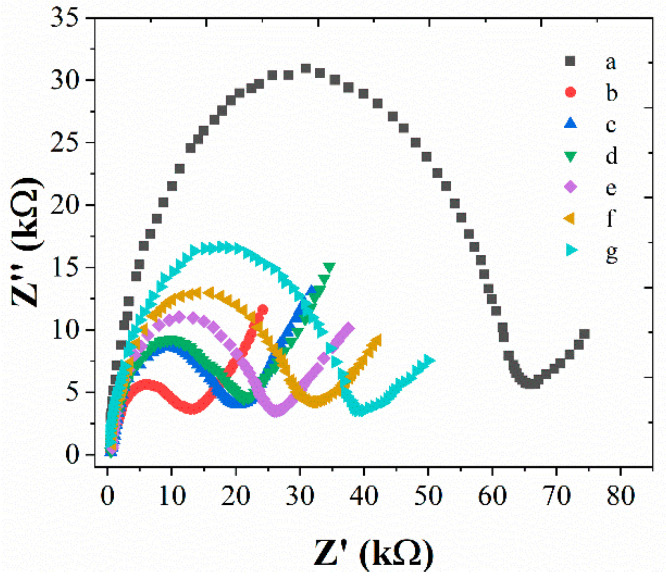
EISs of (**a**) bare glassy carbon electrode (GCE); (**b**) Au/GCE; (**c**) anti-*L. brevis*/Au/GCE; (**d**) ILs/anti-*L. brevis*/Au/GCE; (**e**) BSA/ILs/anti-*L. brevis*/Au/GCE; (**f**) *L. brevis*/BSA/ILs/anti-*L. brevis*/Au/GCE; (**g**) HRP-anti-*L. brevis*/*L. brevis*/BSA/ILs/anti-*L. brevis*/Au/GCE in 5 mM [Fe(CN_6_)]^3−/4−^.

Figure 4 shows the EIS behavior changes during the fabrication of immunosensors. Five mM [Fe(CN_6_)]^3−/4−^ was used as a probe. It can be seen from Figure 4, bare GCE showed the highest R_ct_ compared with other electrodes, suggesting the electro-deposition could significantly enhance the electron transfer rate. Then, a clear increase of the R_ct_ was noted after the immobilization of anti-*L. brevis.* It indicates the successful modification. A further increasing of the R_ct_ has been observed with the immobilization of BSA, *L. brevis*, HRP-anti-*L. brevis* and *L. brevis,* suggesting the successful modification of each step. The increase of R_ct_ during the sensor fabrication is due to the formation of barriers during the antibody-antigen reaction [34,35]. On the other hand, the coating of ILs only affects the R_ct_ slightly, suggesting the ILs is an ideal candidate for enhancing the loading performance of the electrode.

**Figure 5:**
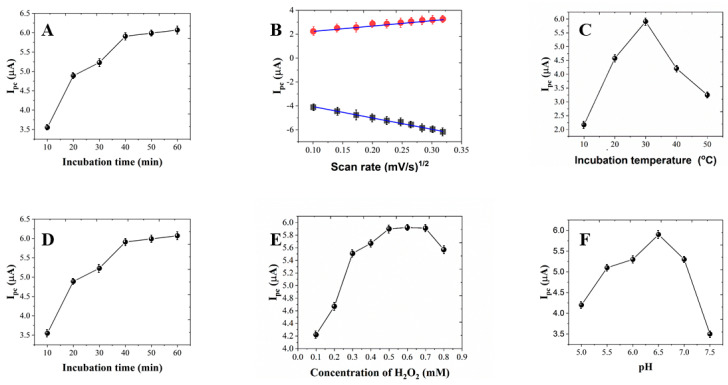
(**A**) Effect of incubation time between *L. brevis* and HRP-anti-*L. brevis* on the immunosensor. (**B**) Current response vs. square root of scan rate (red points and blue points for oxidation and reduction, respectively). (**C**) Effect of incubation temperature on the immunosensor. (**D**) Effect of incubation time between anti-*L. brevis* and *L. brevis* on the immunosensor. (**E**) Effect of concentration of H_2_O_2_ on the immunosensor. (**F**) Effect of pH condition on the immunosensor.

The effect of CV scan rate on the immunosensor can be used to investigate the electron transfer type on the electrode surface. Figure 5A shows the effect of the scan rate of the from 10 to 100 mV/s. It can be seen that the anodic and cathodic peak currents increased linearly with the square root of scan rates. This behavior indicates the immunosensor had a diffusion controlled redox process [16,17,36].

The acidic or alkaline condition can influence the activity of the antibody [9,10,37,38,39]. Figure 5B shows the effect of pH on the immunosensor. It can be seen that the peak current increased along with the pH from 5 to 6.5, and reached the maximum at 6.5. Further increase of pH showed decrease of the current. Therefore, pH 6.5 has been used for sensing.

Figure 5C shows the effect of the H_2_O_2_ concentration on the immunosensor. The increase of the H_2_O_2_ concentration can significantly enhance the sensing performance on the beginning stage due to more H_2_O_2_ participating in the enzymatic reaction [40,41,42]. The current change reached a plateau after 0.5 mM. A decreasing of the current was observed when the concentration exceeded 0.7 mM.

Figure 5D shows the effect of incubation temperature on the immunosensor. It can be seen that the maximum current was observed at 30 ℃. Therefore, 30 ℃ incubation has been used for study. Figure 5E shows the effect of the incubation time between anti-*L. brevis* and *L. brevis* on the immunosensor. The increase of the incubation time can significantly enhance the sensing performance on the beginning stage. The current change reached a plateau after 40 min. Therefore, 40 min incubation has been used for study. Figure 5F shows the effect of the incubation time between *L. brevis* and HRP-anti-*L. brevis* on the immunosensor. Similarly, the increase of the incubation time can significantly enhance the sensing performance on the beginning stage. The current change reached a plateau after 30 min. Therefore, 30 min incubation has been used for study.

**Figure 6:**
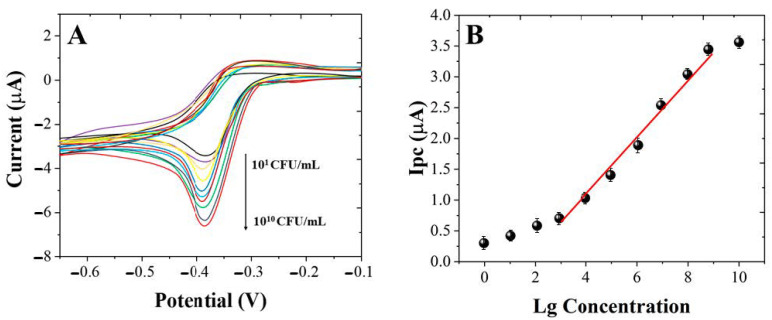
(**A**) CVs of immunosensor towards 10^1^, 10^2^, 10^3^, 10^4^, 10^5^, 10^6^, 10^7^, 10^8^, 10^9^ and 10^10^ CFU/mL of *L. brevis* (an arrow indicates the direction of the increasing *L. brevis* concentration, from 10^1^ to 10^10^ CFU/mL). (**B**) Plots and linear fitting line of ∆_current_ against the concentrates of *L. brevis.*

The sensing performance of the immunosensor was investigated under the optimum conditions. Figure 6A shows the CVs of the immunosensor towards different concentrates of *L. brevis.* As shown in Figure 6B, the ∆_current_ increased along with the the concentrate of *L. brevis* from 10^1^ to 10^10^ CFU/mL. The increase of the ∆_current_ is due to more *L. brevis* being absorbed on the electrode surface, which consequently increased the HRP-anti-*L. brevis* absorption. Then, the HRP-anti-*L. brevis* could catalyze the H_2_O_2_ reduction and contribute to the signal. A linear regression equation can be observed between the ∆_current_ and concentration of *L. brevis* from 10^4^ CFU/mL to 10^9^ CFU/mL. The limit of detection can be estimated to be 10^3^ CFU/mL. Table 1 shows the comparison of proposed immunosensor with previous published works. It can been seen that the immunosensor fabricated in this work showed competitive performance. To further improve the detection sensitivity of the immunosensor, additional probes such as enzyme-assisted catalytic reaction can be included along with the *L. brevis* immobilization in the future work.

**Figure 7:**
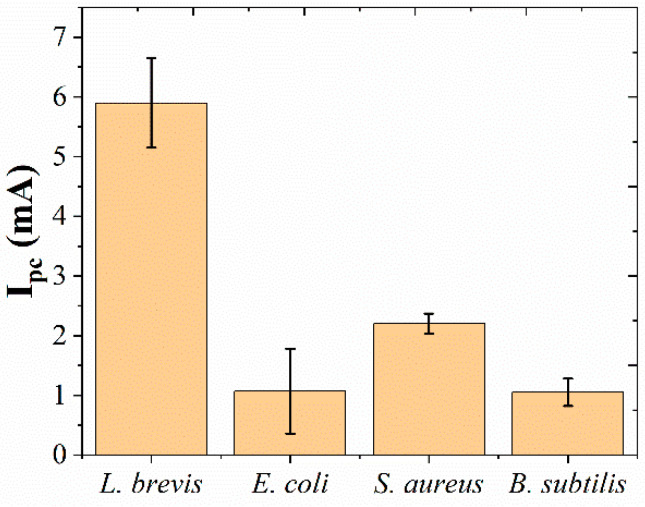
The specificity of immunosensor for *L. brevis*, *E. coli*, *S. aureus* and *B. subtilis*.

The specificity of the immunosensor has been tested using 10^9^ CFU/mL of *E. coli*, *S. aureus* and *B. subtilis*. As shown in Figure 7, the current of immunosensor towards *L. brevis* is significantly larger than that of the sensor towards *E. coli*, *S. aureus* and *B. subtilis*, suggesting the proposed immunosensor had excellent sensing performance. In order to test the use of the proposed immunosensor in beer samples, commercial beer has been tested by replacing the immobilization of *L. brevis*. No ∆_current_ was observed during or after the sensing indicating no detectable *L. brevis* is found in commercial products. Then, standard addition method was applied during the immobilization process. Five individual immunosensors were fabricated using beer mixed with 10^5^ *L. brevis* during the immobilization process. An RSD of 7.21% was detected among the five measurements, suggesting the proposed immunosensor can be applied for sensing *L. brevis* in real beer samples.

## 4. Conclusions

In this work, an ultra-sensitive electrochemical immunosensor was fabricated for *L. brevis* detection. AuNPs were electro-deposited on the electrode surface to enhance the electrochemical performance of the immunosensor. Then, ILs was coated on the immunosensor for enhancing the immobilization performance. Due to the sandwich construction, the proposed electrochemical immunosensor can linear detect *L. brevis* from 10^4^ CFU/mL to 10^9^ CFU/mL. The limit of detection can be estimated to be 10^3^ CFU/mL.

## Figures and Tables

**Table 1:** Comparison of sensing performance towards *L. brevis*.

Sensing Method	Detection Linear Range	Limit of Detection	Reference
Electrochemical sandwich assay	400 to 800 CFU/mL	40 CFU/mL	[43]
Propidium monoazide pretreatment-PCR	10^4^ CFU/mL to 10^8^ CFU/mL	10^4^ CFU/mL	[4]
Electrochemical immunosensor	10^4^ to 10^9^ CFU/mL	10^3^ CFU/mL	This work

## Data Availability

The data presented in this study are available on request from the corresponding author.
